# Supplementary material for: The Longitudinal Effect of Meditation on Resting-State Functional Connectivity Using Dynamic Arterial Spin Labeling: A Feasibility Study
Source: Brain Sci. 2021 Sep 24;11(10):1263. doi: 10.3390/brainsci11101263 (PMC8533789; doi:10.3390/brainsci11101263)
Supplement: Supplementary file 1 [file brainsci-11-01263-s001.zip › brainsci-1326509-supplementary.pdf]

Supplementary Materials

Discussion on relative CBF results

Marginally altered resting CBF was found following 2-month meditation practice using the dASL technique. The resting CBF marginally increased in the junction of the occipital, temporal and parietal regions but decreased in the thalamus region after 2-month meditation practice. The resting CBF in the adjacent occipital-temporal-cerebellum junction region exhibited a marginally negative association with meditation practice time.

A cross-sectional study using continuous arterial spin labeling (CASL) reported the decreased CBF in the occipital/parietal region and increased CBF in the limbic region during the meditative state relative to the control state [56]. Our longitudinal study generally agrees with the locations with trait-related CBF changes induced by meditation, although we observed the opposite polarity of CBF changes, i.e., increased CBF in the occipital/parietal region and decreased CBF in the thalamus/limbic region during rest. Presumably the differences in polarities of CBF changes are dependent on meditation experience because their subjects are experienced meditators (over 30-year meditation experience, approximately 20,000 hours) whereas our subjects are novice meditators (approximately 575 minutes). The occipital CBF increases at rest are also consistent with CBF increases in the region in transcendental meditative status [57]. An EEG study has reported that all meditators (across three types of meditation traditions) exhibited higher parieto-occipital gamma power during meditation than control subjects [58]. A SPECT study reported decreased CBF in the left parietotemporal and occipital gyri during chanting meditation in experienced meditation participants [59]. A PET study reported decreased glucose metabolism during meditative relaxation in experienced meditators [60]. A short-term meditation (5-day training) significantly increased CBF in the occipital lobule of novice meditators [61]. The increased CBF in the occipital region may be associated with an intense degree of visual imagery accompanying the mind wandering process [25,62], especially for novice meditators. Our study also corroborated that the meditation-induced CBF changes are dependent on the practice time of meditation (see Supplementary Fig. 3). After ~1000 minute meditation practice time (cut off time), the CBF changes in the adjacent occipital region become negative, i.e., CBF values in the region are lower than CBF values before practicing the meditation after the cutoff time.

Previous SPECT studies have reported increased CBF in thalamus during the meditative state for experienced Tibetan Buddhist meditators [63] and increased CBF in thalamus during rest for long-term meditators compared to controls [64]. Interestingly, novice meditator with memory loss demonstrated decreased baseline CBF in thalamus after 8-week meditation practice and marginal association of thalamic CBF and neuropsychological scores, although it was not significant after correction of multiple comparisons [65]. The decreased baseline thalamic CBF in novice meditators after 8-week meditation practice agrees with our findings, although their subjects were elderly population whereas ours were young population. Considering the potential regulating role of thalamus in sensory processing and cognitive processes, the altered CBF in thalamus may provide mechanistic support of meditation in improved emotional control, cognitive performance, and sense of focus [66].

Table S1. Summary of cluster-level statistics for clusters showing significant longitudinal CBF changes from the baseline to the follow-up.

	N Voxels	Local Zscore	Peak-t MNI coordinates	Mean beta±std	Anatomical Locations	% Cluster	% Region
Follow-up CBF > Baseline CBF (supplementary Fig.1a)	1497	3.18	-30, -58, 56	0.09±0.03	**Occipital Lobe**		
					Occipital_Mid_L	66.6	30.49
					Occipital_Sup_L	1.80	1.98
					**Temporal Lobe**		
					Temporal_Mid_L	16.43	4.98
					**Parietal Lobe**		
					Parietal_Sup_L	9.42	6.83
					Parietal_Inf_L	4.68	2.86
					Angular_L	1.07	1.36
Follow-up CBF < Baseline CBF (supplementary Fig.1b)	2297	3.62	42, -48, -28	0.06±0.01	**Basal Ganglia**		
					Thalamus_R	20.81	45.22
					Thalamus_L	13.67	28.55
					**Cerebelum**		
					Cerebelum_4_5_L	10.32	21.07
					Cerebelum_4_5_R	4.40	11.73
					Cerebelum_6_L	3.00	4.07
					Cerebelum_6_R	1.26	1.62
					**Occipital Lobe**		
					Lingual_L	9.58	10.50
					Calcarine_L	6.79	6.91
					Fusiform_R	4.75	4.33
					Lingual_R	1.65	1.65
					**Limbic System**		
					Hippocampus_R	5.83	14.16
					ParaHippocampal_R	5.44	11.04
					ParaHippocampal_L	3.61	8.49
					Hippocampus_L	1.96	4.83
					**Parietal Lobe**		
					Precuneus_L	3.00	1.96
CBF change associated with practice time (supplementary Fig. 2)	1101	3.50	-24, -86, 28	(-1.95±0.56)*e-4	**Occipital Lobe**		
					Occipital_Mid_L	54.11	19.54
					Occipital_Sup_L	22.18	19.18
					Occipital_Inf_L	4.32	5.42
					Fusiform_L	3.47	1.77
					**Cerebelum**		
					Cerebelum_Crusl_L	2.79	1.27
					**Temporal Lobe**		
					Temporal_Mid_L	1.61	0.38

MNI = Montreal Neurological Institute.

Table S2. Summary of Post-hoc regional analysis results on the association between rsFC changes and practice time.

Contrast	Seed ROI	Peak-t MNI coordinates	rho	p value
Positive association	PCC	-54, 4, -10	0.97	8.17e-06
		-4, -42, -12	0.93	2.30e-04
		44, 22, -10	0.97	2.37e-05
		44, -24, 18	0.96	5.17e-05
	PFC	8, 36, 14	0.96	5.91e-05
		44, -26, 2	0.97	2.16e-05
		-2, -58, 18	0.91	7.54e-04
	RMT	44, 8, 44	0.92	2.88e-04
		-50, 28, 28	0.92	4.13e-04
		46, 50, -8	0.92	5.02e-04
	RFE	-24, 18, 60	0.95	6.42e-05
	LSPL	-38, 34, 12	0.93	3.47e-04
	LFE	40, -56, 46	0.92	3.81e-04
		26, 20, 64	0.94	1.39e-04
Negative association	PCC	-26, -52, 42	-0.97	1.05e-05
	PFC	36, 34, 18	-0.96	5.95e-05
		22, -68, 52	-0.95	1.15e-04
	RMT	28, -68, 22	-0.93	3.01e-04
		-14, -78, 24	-0.91	5.83e-04
	RFE	0, 32, 24	-0.96	3.19e-05
	LSPL	-2, -64, 4	-0.99	1.24e-06

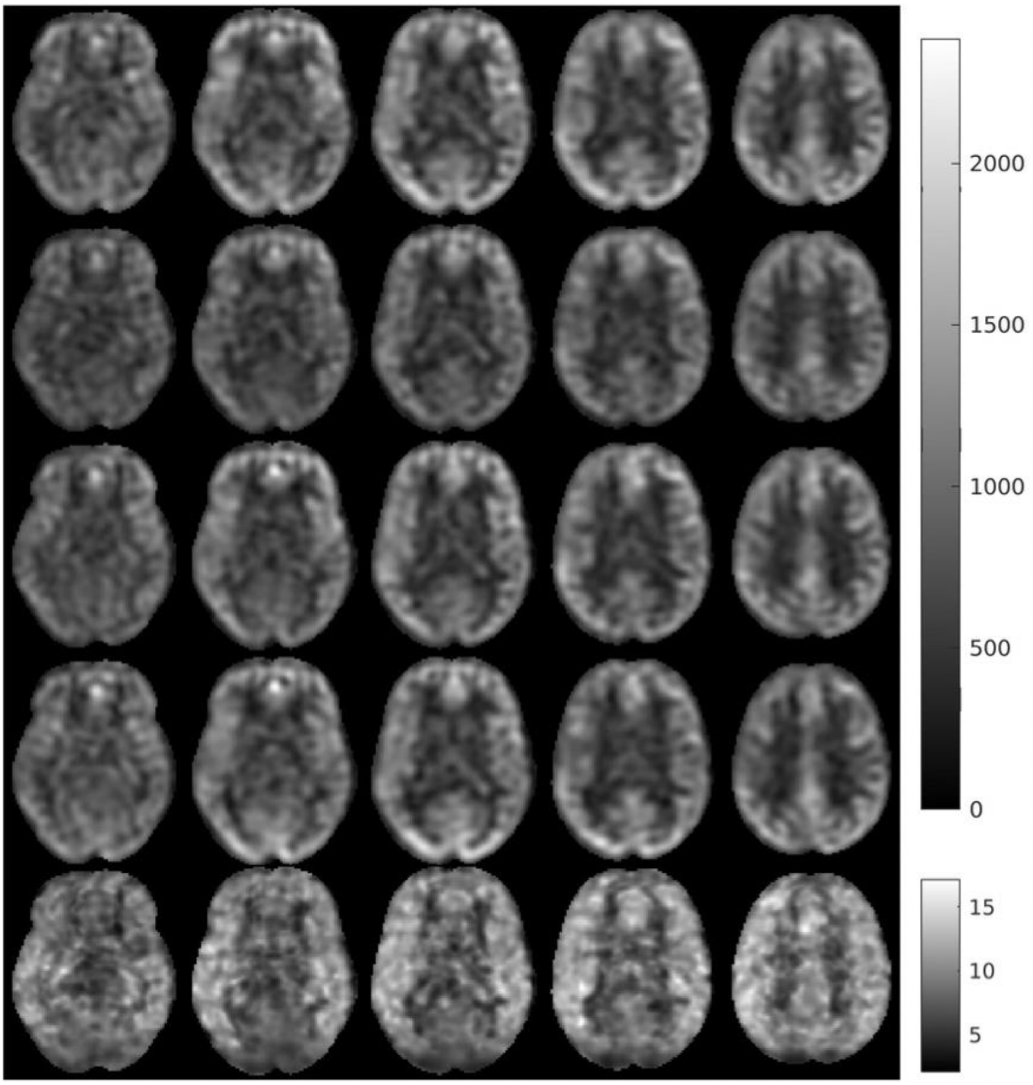

Figure S1. Representative dASL images from the inferior to superior slice (column 1 to 5) at four time points (row 1 to 4) and temporal SNR across all time points (row 5). The scale bars are shown on the right..

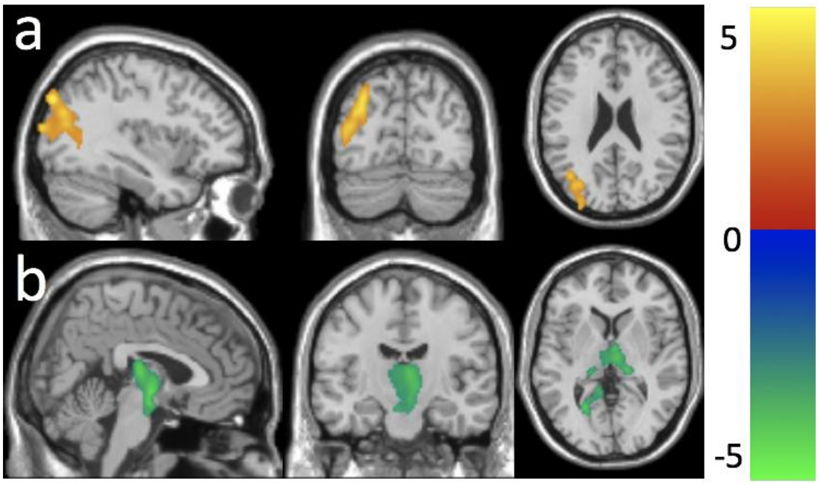

Figure S2. Regions overlaid on a standard brain template in which (a) follow-up CBF > baseline CBF, (b) follow-up CBF < baseline CBF at the thresholds of a voxel-level p-value of 0.01 and corrected cluster-level p-value of 0.05. The color bar shows the range of t-values.

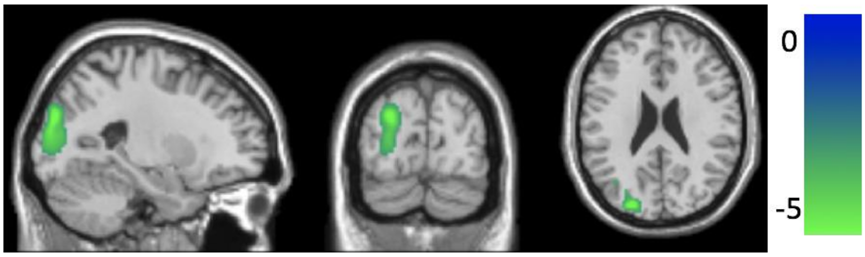

Figure S3. Regions overlaid on a standard brain template in which the meditation practice time was negatively associated with CBF changes at the threshold of a voxel-level p-value of 0.01 and corrected cluster-level p-value of 0.05. Color bar shows the range of t-values.

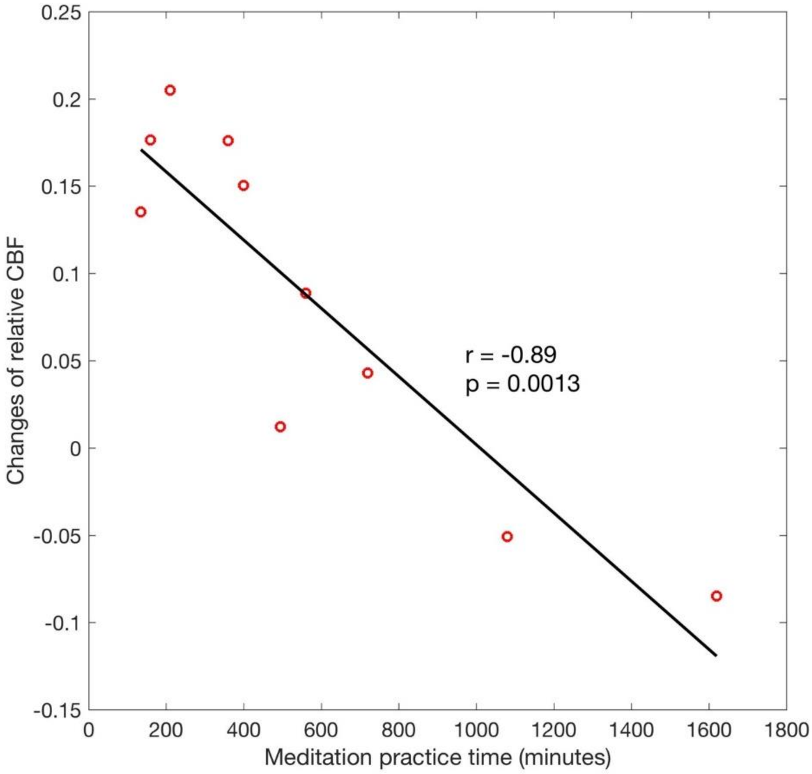

Figure S4. Meditation practice time was negatively associated with relative CBF in the junction of occipital, temporal, and cerebellum regions (r = -0.89, p = 0.0013).

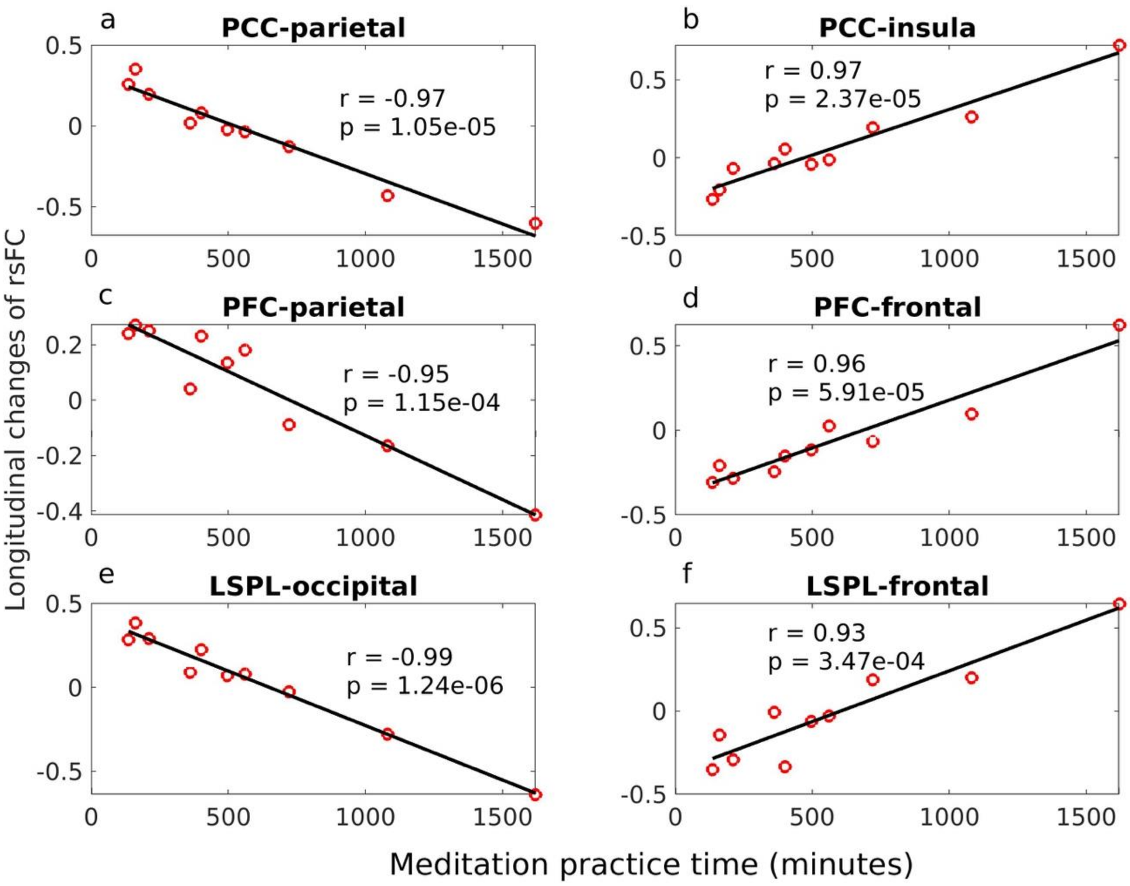

Figure S5. Post-hoc regional analysis confirms that the association of meditation practice time with longitudinal changes of (a) PCC-parietal rsFC, (b) PCC-insula rsFC, (c) PFC-parietal rsFC, (d) PFC-frontal rsFC, (e) LSPL-occipital rsFC, and (f) LSPL-frontal rsFC. The association between longitudinal changes of rsFC and meditation practice time appears not driven by outliers.
